# Predictors of Quality of Life in Portuguese Obese Patients: A Structural Equation Modeling Application

**DOI:** 10.1155/2014/684919

**Published:** 2014-02-16

**Authors:** Estela Vilhena, José Pais-Ribeiro, Isabel Silva, Helena Cardoso, Denisa Mendonça

**Affiliations:** ^1^Polytechnic Institute of Cávado and Ave, 4750-810 Barcelos, Portugal; ^2^ICBAS, University of Porto, 4050-313 Porto, Portugal; ^3^ISPUP, Institute of Public Health, University of Porto, 4050-600 Porto, Portugal; ^4^Faculty of Psychology and Educational Sciences, University of Porto, 4200-135 Porto, Portugal; ^5^UIPES, 1140-041 Lisbon, Portugal; ^6^University of Fernando Pessoa, 4249-004 Porto, Portugal; ^7^UMIB/ICBAS and Hospital Santo António/CHP, 4099-001 Porto, Portugal

## Abstract

Living with obesity is an experience that may affect multiple aspects of an individual's life. Obesity is considered a relevant public health problem in modern societies. To determine the comparative efficacy of different treatments and to assess their impact on patients' everyday life, it is important to identify factors that are relevant to the quality of life of obese patients. The present study aims to evaluate, in Portuguese obese patients, the simultaneous impact of several psychosocial factors on quality of life. This study also explores the mediating role of stigma in the relationship between positive/negative affect and quality of life. A sample of 215 obese patients selected from the main hospitals in Portugal completed self-report questionnaires to assess sociodemographic, clinical, psychosocial, and quality of life variables. Data were analysed using structural equation modeling. The model fitted the data reasonably well, CFI = 0.9, RMSEA = 0.06. More enthusiastic and more active patients had a better quality of life. Those who reflect lower perception of stigma had a better physical and mental health. Partial mediation effects of stigma between positive affect and mental health and between negative affect and physical health were found. The stigma is pervasive and causes consequences for psychological and physical health.

## 1. Introduction 

Obesity is defined as a complex and multifactorial condition affected by interaction of genetic, metabolic, social, behavioral, and cultural factors [1]. Obese people experience impairments resulting in a significant impact on health and contribute to reduced quality of life (QoL) [2, 3]. An individual is considered obese if he/she has a BMI (Body Mass Index) of 30 or more [4]. The interest in studying QoL in these obese patients continues to grow [1]. QoL is a multidimensional construct which is often measured as a subjective assessment of different life domains [5]. The identification of a set of simultaneous factors that contribute to a better quality of life can help to determine the comparative efficacy of different treatments and to assess the impact of treatment on patients everyday life [3]. Although, according to Vieira et al. [3], this type of investigations has not been the focus of many prior research.

Fontaine and Cheskin [6], in their study, consider dispositional optimism as a predictor of weight loss. Dispositional optimism defined as the expectation or belief in positive outcomes in the future [7] has been shown to be associated positively with physical well-being [8]. However, there are few studies where the role of dispositional optimism in QoL in obese patients is studied. In the literature and in consonance with Carr and colleagues [9] the impact of body physical health on quality of life has been broadly documented. Although the relation between physical function limitation and negative affect has not been fully studied in obese people, studies suggest that other chronic physical conditions interfere in functioning of these patients, which in turn triggers negative affect. The authors refer that the obese people have personal characteristics that may affect their mood and contribute to poor health or interpersonal discrimination. Studies reveal that the impact of obesity on feelings and emotions is associated with changes in negative affect rather than in positive affect [9, 10]. However, the relationship between positive and negative affect with QoL remains unclear [10]. According to Wiczinski and colleagues [11] obesity has been shown to be associated with a reduced QoL. These authors also refer that social support may play an important role in QoL. They found that social support was associated with mental and physical QoL components. However, Andenaes and colleagues [2] refer that social support has rarely been studied in obese people.

For obese people, the stigma is generalized and represents numerous consequences for their psychological and physical health [12]. These persons are highly susceptible to institutional and interpersonal discrimination, teasing and, problematic relationships with family members [9, 10]. According to Ogden and Clementi [13] obesity influences not only individuals health but also their psychological state. In obese people with appropriate support, stigma may present the sufficient triggers to encourage the changes which are necessary for weight loss and improve their QoL.

Although these psychosocial factors identified as predictors of QOL are linked and not easily separated, often they have been analysed individually. There is a lack of studies that analyse the simultaneous association between psychosocial predictors and QOL. Then, it will be relevant to examine the simultaneous impact of them on QOL.

The aim of the present study was to evaluate the simultaneous impact of dispositional optimism, positive and negative affect, stigma, and social support factors on quality of life, in Portuguese obese patients. To explore the complexity of the relationship between variables we use structural equation model (SEM) that “is the only analysis that allows complete and simultaneous tests of all the relationships” [14, page 679].

**Figure 1:**
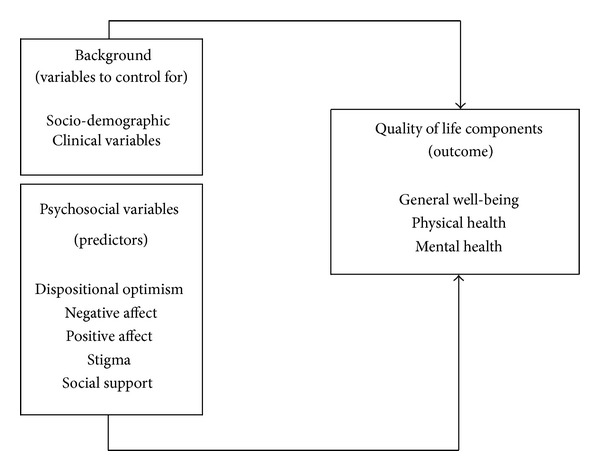
Conceptual model of sociodemographic, clinical, and psychosocial factors influencing QoL.

We have constructed a model (Figure 1) in which we described the influence of psychosocial variables on QoL, controlling for socio-demographic and clinical variables. We hypothesized that psychosocial variables have a simultaneous impact on QoL. It was suggested that the negative effect of stigma is dependent in part on the internal perceptions, beliefs, and emotions of the stigmatized person, above and beyond the effects of direct discrimination by others [15]. Then we hypothesized that stigma exerts a mediator effect between positive/negative affect and QoL components.

## 2. Methods

### 2.1. Sample

This cross-sectional study used a sequential sample of 215 volunteer obese patients. These patients were approached directly by their physicians during the consultation in outpatient departments of four central Portuguese Hospitals (a hospital located in the central coast of Portugal, Lisbon, and four on the north coast, Oporto). All patients agree to collaborate with the help of 5 physicians. Inclusion criteria are as follows: (1) diagnosis of obesity, disease diagnosed at least 3 years prior to the study; (2) age ≥ 17 years at the time of the interview; (3) educational level higher than 6 years; (4) to return to usual daily life with disease under control; (5) no cognitive disturbances. Prior to data collection, ethical approval for this study was obtained from the institutions' ethical committees. After a description of the study aims and the participant rights, all patients who met the inclusion criteria agreed to participate.

### 2.2. Measures

Obese patients completed self-report questionnaires to assess sociodemographic, clinical, psychosocial, and quality of life variables. Psychologists were responsible for data collection after medical appointment. They were trained in the protocol used.

#### 2.2.1. Sociodemographic and Clinical Variables

Data regarding age, sex, education, severity of disease perception (“generally, how do you classify your illness?” coded using an increasing scale from 1: nothing serious to 11: very serious), and time since obesity diagnosis were analised. Severity of disease was assessed with an anchoring vignette scale [16] following the recommendations of Sen [17] and the Eurostat statistics report practices. The scale is similar to the pain severity scale [18].

#### 2.2.2. Psychosocial Variables

*(1) Dispositional Optimism*. Dispositional optimism was evaluated with the Life Orientation Test-Revised (LOT-R) [19]. The LOT-R was developed to assess individual differences in generalized optimism versus pessimism. The Portuguese validated scale [20] showed similar characteristics to the original version. It consists of ten statements, in which three items reflect expectations for positive outcomes, three for negative outcomes, and four are filter items. The optimism score was calculated by adding the three optimism questions value and the pessimism score was calculated by adding the three pessimism questions value. The overall LOT-R score was calculated by reverse scoring the three pessimism scores and summing responses to all six questions. Higher scores indicate greater optimism. The Portuguese version shows a Cronbach *α* of 0.71.

*(2) Positive Affect and Negative Affect*. To assess positive affect (PA) and negative affect (NA), the validated Portuguese version [21] of the Positive and Negative Affect Schedule (PANAS) scale, constructed by Watson et al. [22], was administered. It consists of twenty statements, in which ten items reflect expectations for PA and ten for NA. Items were averaged to obtain scale scores, and higher scores of PA indicate more positive affect or the extent to which the individual feels enthusiastic, active, and alert. A higher score of NA indicates more negative affect, which reflects the individual aversive mood states and general distress. The authors of PANAS calculated the Cronbach *α* coefficients in different samples and found that they ranged from 0.90 to 0.96 for PA and from 0.84 to 0.87 for NA. Portuguese version shows similar characteristics to the original, with a Cronbach *α* of 0.86 for the positive affect and 0.89 for the negative affect scales.

*(3) Stigma*. Self-perception of stigma was assessed using a five-item one-dimensional questionnaire, answered in a likert type scale with seven alternatives between totally agree and totally disagree, developed by Pais-Ribeiro et al. [23]. Higher scores reflect lower perception of stigma. For the five items Cronbach *α* shows a value of 0.82.

*(4) Social Support*. Social support was assessed with the Social Support Survey (MOS) [24, 25]. This is a multidimensional self-questionnaire, adapted to the Portuguese population, that evaluates various dimensions of social support. The MOS consists of four separate social support subscales: emotional/informational, tangible, affectionate, and positive social interaction. An overall functional social support index is also used. All subscales have shown strong reliability over time with a Cronbach *α* higher than 0.91.

#### 2.2.3. Outcome Variables

*(1) Quality of Life*. The 36-item Short-Form Health Survey (SF-36) [26], developed for the MOS study, was used and divided into eight dimensions that represent two major components: the physical and the mental components of health. In this study, we used the results from the IQOLA project [27], in which a second-order factor was found, with three components of SF-36 (general well-being—GWB, physical health—PH, and mental health—MH). All scales and the component scores are positively scored so that higher scores represent better health-related QoL. The Portuguese version of the MOS SF-36 [28, 29] shows a Cronbach *α* of 0.70.

### 2.3. Statistical Analysis

Descriptive statistics were used to describe sociodemographic and clinical characteristics of the sample.

#### 2.3.1. Structural Equation Modeling (SEM)

Structural equation modeling (SEM) was used to test the conceptual model to evaluate the simultaneous impact of dispositional optimism, positive and negative affect, stigma, and social support factors on quality of life. SEM is a multivariate technique that allows for representing, estimating and testing theoretical models that involve several relationships between variables (observed and latent), in order to understand the patterns of correlation/covariance between them [14]. Latent variables are not directly observed, generally they correspond to hypothetical constructs or factors which are explanatory variables presumed to reflect a continuum that is not directly observable [14, 30]. SEM is a combination of factor and path analyses, corresponding to the measurement and structural models, respectively. First, we applied confirmatory factor analysis (CFA) (measurement model) in order to assess whether all the latent variables were represented by their respective indicators (observed variables). The structural model indicates the direct and indirect effects of latent and observed variables (which are not indicators of latent variables). Before estimating the hypothesized model the distribution of continuous variables was analised to assess significant departure from normality. To account for the nonnormality of the data the robust maximum likelihood estimation procedure was used [31]. The adequacy of the model was assessed according to goodness of fit indexes. The Satorra-Bentler Scale chi-square test was used as an index of discrepancy between the original correlation matrix and the correlation matrix estimated from the model [32]. A nonsignificant *P* value (*P* > 0.05) and the ratio (*S* − *B*_*χ*^2^_)/*df* < 3 would represent a good model fit. As the significance of a chi-square test is dependent on the number of subjects, other goodness-of-fit indexes were also used. Comparative Fit Index (CFI), with maximum value 1.00, is derived from the comparison of the hypothesized model with the independent model; a value of CFI > 0.90 suggests a close fit [33]; Root Mean Square Error Approximation (RMSEA) [34] values help to answer the question of how well the model would fit the population covariance matrix if it were available; values less than 0.05 indicate a good fit, being acceptable values until 0.08 [14, 30, 35–37]. Based on multivariate Lagrange Multiplier (LM) tests, post-hoc modifications to the proposed model were made to add new paths as necessary. To compare two or more models the Akaike Information Criterion (AIC) [38] was used, with smaller values representing better model fit. The significance of all direct and indirect effects was evaluated to determine which variables have a direct and indirect impact on QoL. The R^2^ values were calculated for all predictors, mediators, and outcome variables to determine the proportion of explained variance in outcome [30].

**Figure 2:**
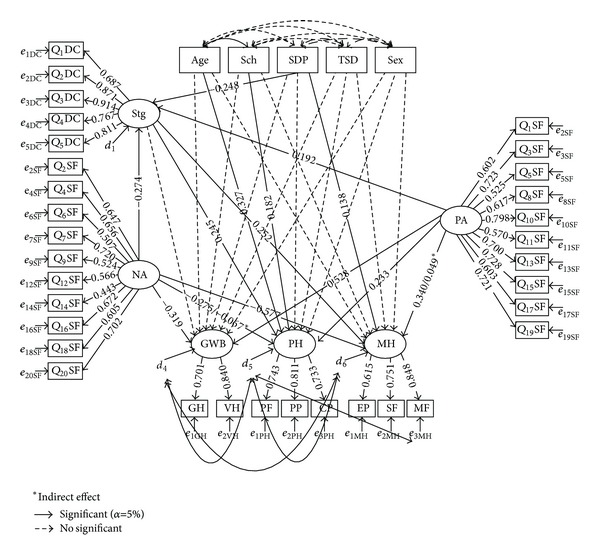
Standardized estimates: Structural Equation Model. Age: age; Sch: education; SDP: severity of disease perception; TSD: time since diagnosis; Sex: sex; Stg: stigma; NA: negative affect; PA: positive affect; GWB: general well-being; PH: physical health; MH: mental health.

In Figure 2 rectangles represent observed variables and circles represent latent variables; the error terms of observed variables are represented by *e* and of latent variables are represented by *d* (disturbances); single-headed arrows represent the influence of one variable in another, and double-headed arrows represent associations between pairs of variables. Analyses were conducted with the EQS 6.1 [39] package and a level of significance of 0.05 was considered.

The observed and latent variables evaluated are summarized in Table 1.

#### 2.3.2. Mediation Analysis

The possible mediation of stigma between positive/negative affect and QoL components was assessed based on the traditional method proposed by Baron and Kenny [40]. A mediating variable transmits the effect of an independent variable on a dependent variable [40, 41]. The analysis of this effect requires several steps: (1) positive (negative) affect is a predictor of a QoL component, (2) a positive (negative) affect is a predictor of stigma, (3) controlling for stigma, the relationship between a positive (negative) affect and a QoL component should reduce or cease to be statistically significant. If it fails to be statistically significant, then we have a full mediation model (i.e., mediator explains completely the relationship between a psychosocial variable and QoL component). However, if the relationship between a positive (negative) affect and QoL component decreases significantly, we have a partial mediation. Significance of the mediation is based on the indirect effect. The indirect effect may be estimated in two ways, either $\hat{a}\cdot \hat{b}$ or $\hat{c}-\hat{c^{\prime }}$, where *c* is the regression coefficient of the model regressing the QoL component on positive (negative) affect *a* is the regression coefficient relating positive (negative) affect and stigma, *b* is the regression coefficient relating stigma and the QoL component adjusted for positive (negative) affect, and *c*′ is the coefficient relating positive (negative) affect and QoL component but now adjusted for stigma. The value of the mediated or indirect effect estimated by taking the difference in the coefficients, $\hat{c}-\hat{c^{\prime }}$, corresponds to the reduction in the independent variable (positive/negative affect) effect on the dependent variable (QoL components) when adjusted for the mediator (stigma). To test for significance, the difference is then divided by the standard error of the difference and the ratio is compared to a standard normal distribution [41].

## 3. Results

### 3.1. Sample Characteristics

The mean age of patients was 42.98 years (sd = 11.3) and 86.5% were female. Mean level education was 8.17 years (sd = 4.19), mean time since diagnosis was 11 years (sd = 9.5), and mean perception of severity of disease was 7.31 (sd = 2.69).

### 3.2. Analysis

First, the individual impact of each psychosocial factor in QoL components, controlling for sociodemographic and clinic variables, was analised. Then, the simultaneous impact of dispositional optimism, positive and negative affect, stigma and social support factors on QoL components was analysed, evaluating the hypothetical model postulated in the aim of the study.

Tables 2 and 3 show the results obtained for the measurement and structural models, for the first analysis where each psychosocial factor was independently analysed.

For all psychosocial factors, the results suggest a satisfactory model fit. All factor loadings between each indicator and latent variables were statistically significant, indicating that all were well represented by their respective indicators. The proportion of explained variance for each indicator was moderate to high.

The structural modelfit statistics indicate an acceptable model fit for all psychosocial factors (Table 3). Controlling for sociodemographic and clinical variables, all factors had a statistically significant impact on the components of QoL. Models results showed that an optimist attitude, a good social support, a lower perception of stigma, and more positive affect contribute to better general well-being and better physical and mental health. Just the negative affect behaves like a negative predictor of QoL.

The following results refer to the model where the simultaneous impact of psychosocial variables on quality of life was considered.

Measurement model include eight latent variables (dispositional optimism, negative and positive affect, stigma, social support, general well-being, and physical and mental health) and 43 observed variables referring to the corresponding indicators of the eight latent variables. The results showed a satisfactory model fit: *S* − *B*_*χ*_832_^2^_ = 1271.8896, *P* < 0.001; (*S* − *B*_*χ*^2^_)/*df* = 1.52; CIF = 0.90; RMSEA = 0.054, RMSEA (90% IC) = (0.04; 0.06). All factor loadings between each indicator and latent variables were statistically significant, indicating that all were well represented by their respective indicators. The proportion of explained variance for each indicator was also moderate to high (*R* ^2^ values ranging from 0.22 to 0.87).

According to the objective of this research, our main model analyse the simultaneous impact of dispositional optimism, positive and negative affect, stigma, and social support on QoL controlling for sociodemographic and clinical variables. The mediation effect of stigma between positive/negative affect and the QoL components was also examined (model 1).

However, the social support and dispositional optimism were found not to be statistically significant and were subsequently removed from the model, corresponding to model 2 and model 3, respectively. Results of overall model fit and of the comparison between the three models are shown in Table 4.

The results show that fit indexes are comparable in the three models, although in the model 1 and model 2 the CFI values were in the borderline. Based on the Akaike Information Criterion (AIC) we chose to present model 3. Standardized parameters of model 3 are presented in Figure 2.

The results (Figure 2) showed that age has a negative impact (*b* = −0.327) and school grade has a statistically significant positive impact (*b* = 0.182) on physical health. The severity of disease perception influences statistically and positively the mental health (*b* = 0.138). A simultaneous direct and positive impact between stigma and physical (*b* = 0.245) and mental health (*b* = 0.252) was found. Positive affect had a statistically and positively significant impact on the components of QoL: general well-being (*b* = 0.528), physical health (*b* = 0.233), and mental health (*b* = 0.340). Negative affect had also an impact, but statistically negative, on general well-being (*b* = −0.319), physical health (*b* = −0.275), and mental health (*b* = −0.577).

Mediation analyses were evaluated testing the significance of the indirect effect between predictors and QoL components [42]. The results showed two statistically significant indirect effects, one meaning that stigma exerts a partial mediation between positive affect and mental health ($\hat{a}\hat{b}=0.05$, *P* < 0.05) and the other that stigma exerts a partial mediation between negative affect and physical health ($\hat{a}\hat{b}=-0.067$,  *P* < 0.05).

## 4. Discussion

The prevalence of obesity is increasing rapidly and becomes a major public health problem in many countries [2]. Portugal is not an exception. The prevalence in Portugal is 15.1%, lower than that reported in the USA (35.9%) [43]. Several successful weight reduction programs have been implemented in Portugal [44, 45].

Our study is concentrated on psychosocial predictors of QoL, which are related and not easily separated. There is a lack of studies that have examined the simultaneous associations between psychosocial predictors and quality of life outcomes. Our study contributes to the reduction of this fact and to better understand the role of psychosocial variables in the quality of life.

The factors identified as predictors of QoL could be helpful for health care and improving the measurement of treatment efficacy and help to assess and compare the efficacy of different treatments. These findings can help to assess the impact on how patients feel and function in their everyday life [1].

Despite the strengths of this study, it is important to note some of its limitations. The cross-sectional design does not allow any conclusions on the longitudinal evaluation of these patients.

Furthermore, this is a hospital-based study and patients attending primary health care units are underrepresented. Although our findings constitute a relevant contribution, further studies are needed to better address the topic of QoL in obese patients.

The primary goal of this study was to identify the psychosocial predictors of QoL in a Portuguese obese patients. To briefly summarize our findings, controlling for sociodemographic and clinical variables, we found that positive and negative affect and the stigma factors were relevant predictors of QoL.

In this study, first the individual impact of dispositional optimism, positive and negative affect, stigma, and social support factors on QoL components was analysed, in order to compare our findings with those from other studies with similar approaches. The results are consistent with the literature [2, 8–10], showing that a more optimistic attitude, a better positive affect, a lower perception of stigma or a better social support contribute to general to a better QoL.

However the principal goal of the study was to take into account simultaneous psychosocial variables, with the aim to clarify their simultaneous impact on QoL. The dispositional optimism and the social support have been referred as important factors in the life of obese individuals. In this study their impacts on QoL cease to be significant in presence of positive and negative affect and stigma factors.

Research from Pasco et al. [46] and Carr et al. [47] shows that obese persons have significantly higher levels of negative affect than their thinner peers. Also a systematic review [48] shows that a quarter of the population in Germany displayed definite stigmatizing attitudes about obesity. Similar results were found by Sutin and Terracciano [49] about negative attitudes toward obese people in the American society resulting in poorer mental health outcomes and that weight discrimination increases risk for obesity. In concordance with these findings our study suggests that negative attitudes toward obese people or stigma can result in poor health outcomes.

Puhl and Heuer [50] report that stigma and discrimination toward obese persons are pervasive and pose numerous consequences for their psychological and physical health.

Similarly, Schafer and Ferraro [51] reported that obesity is widely recognized as a health risk, representing a disadvantaged social position. They also refer that perceived weight discrimination is harmful and increases the health risks of obesity. Puhl et al. [52], also show that the language used by health professionals expresses negative attitudes toward overweight and obese people, and has negative impacts on obese people.

This study reveals that positive affect has a statistically significant and positive impact on all components of QoL. More enthusiastic and active people have a better subjective well-being and a better physical and a better mental health. Negative affect behaves like a negative predictor of physical and mental health. Aversive mood states contribute to a poor QoL. Other researches [9, 10] support that the positive and negative affect reveal that obese patients are more likely to have negative affect and have more negative feelings such as distress, anger, fear, and shame.

Obese people with lower perception of stigma have a better quality of life, in physical and mental domains. Vartanian and Smyth [53] refer that several antiobesity campaigns appear to embrace stigmatization of obese individuals as a public health strategy. Those campaigns are based in the idea that stigmatizing obese individuals will motivate them to change their behavior and will also result in successful behavior changes. Puhl and Heuer [12] refer that stigmatization of obese patients represents serious risks to physical health. Stigma seems to have potential costs and benefits in obesity.

The results of this study also showed the relation between negative affect and physical health where mediated by stigma, as well as the relation between positive affect and mental health. These results support the importance of the stigma in QoL of these patients.

The use of SEM allows us to understand the complexity of the simultaneous relationships between the variables we use. The study suggests that all the variables are important but when taken together they can have different levels of importance for the explanation of the results.

## Figures and Tables

**Table 1:** Observed and latent variables in the assessment of quality of life of obese people.

Latent variables	Observed variables
Psychosocial variables	
Dispositional optimism (DO)	*Q*_*i*_ Life orientation *i* = 1,…, 6
Negative affect (NA)	*Q*_*i*_ Scale of feelings *i* = 1,…, 10
Positive affect (PA)	*Q*_*i*_ Scale of feelings *i* = 1,…, 10
Stigma (Stg)	*Q*_*i*_ disease condition *i* = 1,…, 5
Social support (SS)	Affective (SSA)
	Tangible (SST)
	Positive social interaction (SSPSI)
	Emotional/informational (SSEI)

QoL components	
General well-being (GWB)	General health (GH)
	Vital health (VH)
Physical health (PH)	Physical function (PF)
	Physical pain (PP)
	Corporal pain (CP)
Mental health (MH)	Emotion pain (EP)
	Social function (SF)
	Mental function (MF)

**Table 2:** Goodness-of-fit test for measurement model.

Measurement model						
Predictor variable	*S* − *B*_*χ*^2^_	df	*P*	(*S* − *B*_*χ*^2^_)/df	CFI	RMSEA (90% IC)
Optimism	144.0097	71	<0.001	2.03	0.936	0.071 (0.05, 0.08)
Social support	101.3522	48	<0.001	2.11	0.959	0.074 (0.05, 0.09)
Stigma	145.8685	59	<0.001	2.47	0.949	0.085 (0.06, 0.10)
Positive affect	191.4264	129	<0.001	1.48	0.958	0.050 (0.03, 0.06)
Negative affect	270.2263	129	<0.001	2.09	0.913	0.075 (0.06, 0.08)

**Table 3:** Goodness-of-fit test for structural model.

Predictor variable	Structural model						
	*b*	*S* − *B*_*χ*^2^_	df	*P*	(*S* − *B*_*χ*^2^_)/df	CFI	RMSEA (90% IC)
Optimism		230.71	128	<0.001	1.80	0.913	0.065 (0.05, 0.07)
GWB	0.61						
PH	0.32						
MH	0.48						
Social support		170.048	95	<0.001	1.78	0.944	0.064 (0.05, 0.08)
GWB	0.48						
PH	0.38						
MH	0.52						
Stigma		232.9195	111	<0.001	2.09	0.930	0.076 (0.06, 0.08)
GWB	0.50						
PH	0.51						
MH	0.64						
Positive affect		312.2503	205	<0.001	1.52	0.933	0.053 (0.04, 0.06)
GWB	0.67						
PH	0.39						
MH	0.54						
Negative affect		365.7437	205	<0.001	1.78	0.902	0.066 (0.05, 0.07)
GWB	−0.54						
PH	−0.44						
MH	−0.76						

**Table 4:** Structural equations models and model fit indexes.

Model fit indexes							
Model	*S* − *B*_*χ*^2^_	df	*P*	(*S* − *B*_*χ*^2^_)/df	CFI	RMSEA (90% IC)	AIC
1	1719.94	1039	<0.001	1.65	0.83	0.062 (0.057, 0.067)	2755.13
2	1453.50	864	<0.001	1.68	0.83	0.063 (0.057, 0.069)	2491.79
3	1006.34	629	<0.001	1.59	0.90	0.059 (0.052, 0.065)	2375.35
